# Antidepressants during and after Menopausal Transition: A Systematic Review and Meta-Analysis

**DOI:** 10.1038/s41598-020-64910-8

**Published:** 2020-05-15

**Authors:** Ching-Kuan Wu, Ping-Tao Tseng, Ming-Kung Wu, Dian-Jeng Li, Tien-Yu Chen, Fu-Chen Kuo, Brendon Stubbs, Andre F. Carvalho, Yen-Wen Chen, Pao-Yen Lin, Yu-Shian Cheng, Cheuk-Kwan Sun

**Affiliations:** 1 Department of Psychiatry, Tsyr-Huey Mental Hospital, Kaohsiung Jen-Ai’s Home, Kaohsiung, Taiwan; 2 WinShine Clinics in Specialty of Psychiatry, Kaohsiung City, Taiwan; 3 Department of Psychiatry, Kaohsiung Chang Gung Memorial Hospital and Chang Gung University College of Medicine, Kaohsiung, Taiwan; 4 Graduate Institute of Medicine, College of Medicine, Kaohsiung Medical University, Kaohsiung, Taiwan; 5 Department of Addiction Science, Kaohsiung Municipal Kai-Syuan Psychiatric Hospital, Kaohsiung City, Taiwan; 6 Department of Psychiatry, Tri-Service General Hospital; School of Medicine, National Defense Medical Center, Taipei, Taiwan; 7 Institute of Brain Science, National Yang-Ming University, Taipei, Taiwan; 8 Department of Obstetrics & Gynecology, E-Da Hospital, Kaohsiung, Taiwan; 9 School of Medicine, College of Medicine, I-Shou University, Kaohsiung, Taiwan; 10 Physiotherapy Department, South London and Maudsley NHS Foundation Trust, London, UK; 11 Department of Psychological Medicine, Institute of Psychiatry, Psychology and Neuroscience (IoPPN), King’s College London, De Crespigny Park, London, UK; 12 Faculty of Health, Social Care and Education, Anglia Ruskin University, Chelmsford, UK; 13 Department of Psychiatry, University of Toronto, Toronto, ON Canada; 14 Centre for Addiction & Mental Health (CAMH), Toronto, ON Canada; 15 Prospect Clinic for Otorhinolaryngology & Neurology, Kaohsiung, Taiwan; 16 Institute for Translational Research in Biomedical Sciences, Kaohsiung Chang Gung Memorial Hospital, Kaohsiung, Taiwan; 17 Department of Emergency Medicine, E-Da Hospital, Kaohsiung, Taiwan; 18 Institute of Biomedical Sciences, National Sun Yat-sen University, Kaohsiung, Taiwan; 19 Department of Chemical Engineering and Institute of Biotechnology and Chemical Engineering, I-Shou University, Kaohsiung, Taiwan; 20 Institute of Medical Science and Technology, National Sun Yat-sen University, Kaohsiung, Taiwan

**Keywords:** Health care, Medical research, Psychiatric disorders

## Abstract

To assess the therapeutic benefits of antidepressants in depressive women during and after menopausal transition, PubMed, Cochrane Library, EMBASE and Science Direct were systematically searched from inception to February 1, 2020 for randomized controlled trials examining antidepressants compared to placebo. Primary outcome was change in depressive symptom severity, while secondary outcomes were rates of response/remission rates and dropout/discontinuation due to adverse events. Seven trials involving 1,676 participants (mean age = 52.6 years) showed significant improvement in depressive symptoms (k = 7, Hedges’ g = 0.44, 95% confidence interval (CI) = 0.32 to 0.57, p < 0.001) relative to that in controls. Furthermore, response (k = 3, odds ratio (OR) = 2.53, 95% CI = 1.24 to 5.15, p = 0.01) and remission (k = 3, OR = 1.84, 95% CI = 1.32 to 2.57, p < 0.001) rates were significantly higher in antidepressant-treated groups compared to those with controls. Although dropout rates did not differ between antidepressant and control groups (k = 6, OR = 0.93, 95% CI = 0.70 to 1.26, p = 0.68), the rate of discontinuation due to adverse events was significantly higher in antidepressant-treated groups (k = 6, OR = 0.55, 95% CI = 0.35 to 0.86, p = 0.01). Subgroup analysis indicated that antidepressants were also efficacious for depressive symptoms in those without diagnosis of MDD. The results demonstrated that antidepressants were efficacious for women with depressive syndromes during and after menopausal transition but associated with a higher risk of discontinuation due to adverse events.

## Introduction

Accumulating evidence indicates that women appear to be at a particularly higher risk of the emergence of major depressive disorder (MDD) and also depressive symptoms not severe enough to meet the diagnostic criteria of MDD during menopausal transition^1^. According to the Stages of Reproductive Aging Workshop (STRAW) staging system based on FSH level and regularity of menstrual cycle, the reproductive period of women can be divided into three phases (i.e., reproductive, menopausal transition, and postmenopause) starting from menarche^2^. With the final menstrual period (FMP) being set as the anchor, five stages occur before the FMP (i.e., three stages in the reproductive phase and two stages during menopausal transition), while there are two stages (i.e., early and late) in the postmenopausal phase. The STRAW staging system also indicates an overlapping period of one year of amenorrhea between perimenopause and postmenopause starting from the end of menopausal transition. In recent years, depression during menopausal transition is also considered to be a unique subtype of depression^3^; the accompanying somatic disorders, such as vasomotor symptoms (e.g., hot flashes and night sweats), changes in sexual function^4^, and sleep disturbances^5^, may contribute to the development of depressive symptoms or delay the recognition of affective disorders during this phase of the female reproductive cycle^6^.

The management of depressive symptoms among peri- and post-menopausal women can be challenging. For example, although some guidelines suggested hormone replacement therapy (HRT)^7^, other studies have reported concerns that may limit its widespread use, including controversial therapeutic benefits^8^, a possible increased risk of developing cancer^7,9^, and a high recurrence rate of depressive symptoms following the cessation of HRT^10^. Therefore, antidepressant agents may provide a clinically useful alternative for the management of depressive disorders during menopause. However, the results of previous studies in this population have been inconsistent, with different findings being reported in the beneficial effects of serotonin-norepinephrine reuptake inhibitors (SNRIs)^11^ and selective serotonin reuptake inhibitors (SSRIs)^12^. As a result, the Endocrine Society in the U.S. suggests that antidepressants should only be used for the management of MDD during menopause^7^. Furthermore, depressive symptoms of severity less than that of MDD (i.e., subthreshold depression) have also been reported to be prevalent in this population, and an ever increasing body of evidence indicates that they have a detrimental impact on the quality of life and functioning during menopausal transition^13,14^. Certain guidelines endorsed the use of antidepressants or psychotherapy as frontline treatments for perimenopausal depression^15^. Nevertheless, evidence for the therapeutic benefits of antidepressants for menopausal women with subthreshold depressive symptoms is controversial^16,17^.

Therefore, the current study aimed at providing a comprehensive systematic review and meta-analysis of all randomized controlled clinical trials (RCTs) evaluating the effects of antidepressants in peri- and post-menopausal women with the whole spectrum of depressive disorders during menopausal transition. In addition, we aimed at: (1) assessing the therapeutic effects of antidepressant treatment in this population; (2) evaluating whether potential benefits of antidepressant agents differ in those with full-blown MDD compared to those experiencing subthreshold depression; and (3) investigating the safety and tolerability of antidepressants.

## Methods

### Guidelines and protocol

This systematic review and meta-analysis was conducted according to the guidelines presented in the *Preferred Reporting Items for Systematic Reviews and Meta-Analyses* (PRISMA) statement^18^ (Supplementary Table S1).

### Search strategy and identification of eligible studies

Two investigators (YS Cheng and PT Tseng) independently searched the PubMed/MEDLINE, Cochrane Library, EMBASE and Science Direct electronic databases from inception to February 1, 2020, using the following keywords: (antidepressants and [perimenopause or postmenopause or menopause] and [depression or depressive disorder or mood]). In addition, the ClinicalTrials.gov database was searched using the following search string: ([antidepressant] AND [depression and menopause]). The search of PubMed/MEDLINE was limited to clinical trials. This search strategy was augmented through a manual search of the reference lists of eligible articles as well as relevant clinical guidelines and review articles^1,4,5,7–9,19^.

Two authors (YS Cheng and PT Tseng) screened the titles and abstracts of retrieved references for eligibility. A list of potentially eligible studies was constructed by consensus, after which full-text examinations were conducted. A third reviewer (CK Sun) was consulted if any inconsistencies arose.

### Eligibility criteria

The inclusion criteria were: (1) peer-reviewed articles investigating the efficacy of antidepressants on depressive symptoms in menopausal women meeting the criteria for MDD or experiencing subthreshold depressive symptoms; and (2) articles that were controlled trials conducted in humans. No language restrictions were applied.

The exclusion criteria were: (1) animal studies; (2) trials not related to the treatment effect of antidepressants on depressive symptoms; and (3) studies without a placebo group (i.e., head-to-head trials).

### Methodological quality appraisal

Two independent authors (YS Cheng and PT Tseng) evaluated the risk of bias (inter-rater reliability, 0.85) for each domain described in the Cochrane risk of bias tool^20^.

### Primary outcome

The primary outcome measure was a change in the severity of depressive symptoms as rated by standard instruments used in each included study.

### Secondary outcomes

Secondary outcomes of interest included response and remission rates in each group. Treatment response was defined as a decrease of ≥50% from baseline depression rating scale score as applied in each study. Remission rate was defined as ≤7 points on the Hamilton-depression rating scale score, or ≤10 points on the Montgomery-Åsberg depression rating scale. We also evaluated the safety of the antidepressants considering dropout rates and the rate of discontinuation due to adverse events.

### Data extraction and management

Two independent authors extracted data from the eligible studies into a database of pre-determined variables of interest, including mean age (years), mean body mass index (BMI), duration of antidepressant treatment (weeks), and ethnicity (Caucasian, African American, Hispanic, or Asian). The corresponding authors were contacted by email to request additional data on at least two different occasions 1 week apart whenever variables of interest were not available.

### Statistical analysis

Based on the presumed high heterogeneity among the included studies, data were analyzed using random-effects meta-analysis models rather than fixed effects models^21^ using Comprehensive Meta-Analysis software version 3 (Biostat, Englewood, NJ). Effect sizes (ESs) of changes in depressive symptoms between groups were analyzed using Hedges’ *g* and 95% confidence intervals (95% CIs). We calculated odds ratios (ORs) with 95% CIs for secondary outcomes using dichotomous items (such as response and remission rates).

Heterogeneity was evaluated using the Q statistic^22^, and the *I*^2^ statistic was used to evaluate the proportion of variation^23^. We examined publication bias by visually inspecting funnel plots when less than ten datasets were available^24^, while Egger’s regression test was used when ten or more independent datasets were available^25^. We performed the Duval and Tweedie trim and fill test to adjust ESs when evidence of publication bias was found^26^. With the automated program of Comprehensive Meta-Analysis software version 3, we arranged sensitivity analysis to verify whether an outlier could be biasing our ES estimates^27^. To be specific, to comprehensively evaluate the potential bias contributed to an outlier, we removed one study at a time from the analysis and completed the examination of potential bias contributed by each study included in the current study.

To evaluate potential sources of heterogeneity and confounding effects, we performed meta-regression and subgroup meta-analyses. Specifically, when there were at least ten datasets we conducted the meta-regression procedure using the unrestricted maximum likelihood method. Regarding subgroup meta-analysis, we focused on the studies that used SSRIs or SNRIs as the antidepressant agents, those that only included participants with a diagnosis of MDD; and trials that excluded participants with MDD [i.e., trials that assessed the effects of antidepressants on patients with subthreshold depressive symptoms (defined as those with depressive symptoms not severe enough to meet the diagnostic criteria of MDD)]. Furthermore, we performed subgroup analysis according to the different depression rating scales that were used in the studies. Subgroup analyses were performed when data from at least three independent studies were provided^28^. Statistical significance was set at a two-tailed *alpha* level of 0.05.

## Results

### Study selection

**Figure 1:**
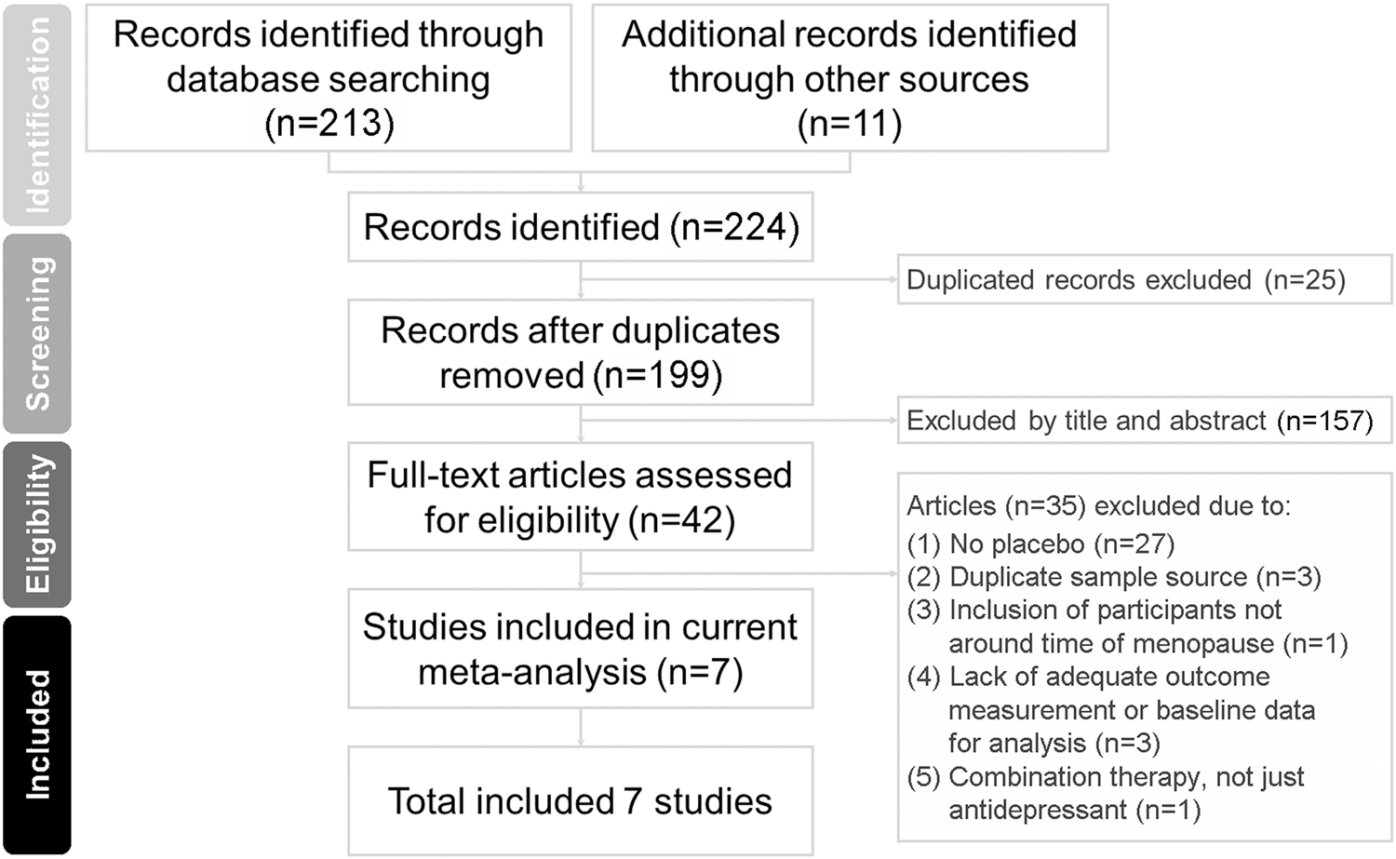
Flowchart of the Current Systematic Review and Meta-analysis.

The PRISMA flowchart used for study selection in this systematic review is shown in Fig. 1. After excluding duplicates, 42 full-text articles were assessed for eligibility. Among them, 35 were excluded due to the following reasons including: (1) No placebo (n = 27), (2) Duplicate sample source (n = 3), (3) Inclusion of participants not around time of menopause (n = 1), (4) Lack of adequate outcome measurement or baseline data for analysis (n = 3), and (5) Combination therapy, not just antidepressant (n = 1) [Supplementary Table S2]. Therefore, seven articles were eligible for the current meta-analysis (Table 1)^11,12,16,17,29–31^.

**Table 1:** Summary of characteristics of studies in the current meta-analysis. STRAW, Stages of Reproductive Aging Workshop; MDD, major depressive disorder; N/A, information not available; DSM-IV, Diagnostic and Statistical Manual of Mental Disorders (Fourth Edition); RCT, randomized controlled trial; BDI, beck depression inventory; HAM-D, Hamilton Depression Rating Scale; GS, Greene Scale; QIDS-SR, quick inventory of depressive symptomatology; MADRS, Montgomery–Åsberg depression rating scale; CGI-S, clinical global impression-severity; CGI-I, clinical global impression-improvement.

Author (year)^Ref^	Menstrual status	Other diagnosis	Criteria	Design	Comparison	N	Duration (weeks)	Outcome	Mean age (years)	Country
Davari- Tanha (2016)^29^	Postmenopausal (STRAW staging system)	N/A	N/A	RCT	Venlafaxine75 mg/d	20	8	BDI (−)	51.0	Iran
					Citalopram 20 mg/d	20				
					Placebo	20				
Macias-Cortes (2015)^12^	Peri-menopausal: Change in cycle length of seven days or longer in either direction from the participant’s own baseline for at least two cycles, or Late transition to menopause (i.e., three to 11 months of amenorrhea) OR Post-menopausal (i.e., 12 months or more of amenorrhea)	MDD	DSM-IV	RCT	Fluoxetine 20 mg/d	39	6	BDI (−) HAM-D (+) GS (−) Response rate (HAM-D) Remission rate (HAM-D)	49.2	Mexico
					Placebo	37			48.8	
Clayton (2013)^11^	Peri- menopausal: The presence of any of the following within 6 months before baseline: 1. Absolute change ≥7 days in menstrual cycle length, or 2. Change in menstrual flow amount (2 or more flow categories), or 3. Change in duration of menses (≥2 days), or 4. Amenorrhea lasting ≥3 months OR Post-menopausal: 1. Amenorrhea >12 months 2. Amenorrhea between 6–12 month with FSH > 40 mIU/mL, or 3. Amenorrhea >6 months post bilateral oophorectomy	MDD	DSM-IV	RCT	Desvenlafaxine 50 mg/d	185	10	HAM-D (+) QIDS-SR MADRS (+) CGI-S Response rate (HAM-D) Remission rate (HAM-D) Response rate (CGI-I)	53.2	USA
					Placebo	178			52.8	
Cheng (2013)^16^	Menopausal: 1. Amenorrhea >12 months 2. Amenorrhea between 6–12 month with FSH > 40 mIU/mL, or 3. Amenorrhea >6 months post bilateral oophorectomy)	N/A	N/A	RCT	Desvenlafaxine100 mg/day	153	12	POMS-TMD GS – depression (+)	53.4	USA
					Desvenlafaxine 150 mg/day	152				
					Placebo	153				
Kornstein (2010)^30^	Peri-menopausal: The presence of any of the following within 6 months of baseline: 1. Absolute change of 7 days or more in menstrual cycle length, or 2. Change in menstrual flow amount (2 or more flow categories, e.g., from light or moderately light to moderately heavy or heavy), or 3. Change in duration of menses (absolute change of 2 or more days); and periods of amenorrhea lasting at least 3 months. OR Post-menopausal: 1. Amenorrhea >12 months, or 2. Amenorrhea >6 months post bilateral oophorectomy)	MDD	DSM-IV	RCT	Desvenlafaxine100–200 mg/d	212	8	HAM-D (+) QIDS-RS MADRS (+) CGI-S CGI-I Response rate (HAM-D) Remission rate (HAM-D) Response rate (CGI-I)	52.0	USA
					Placebo	109			53.0	
Soares (2008)^31^	Peri- or menopausal (STRAW staging system)	N/A	N/A	RCT	Paroxetine 25 mg/day	50	6	MADRS (+)	55.6	Canada
					Placebo	50			57.0	
Suvanto-Luukkonen (2005)^17^	Postmenopausal: Amenorrhea >6 months with FSH > 30 IU/L)	N/A	N/A	RCT	Fluoxetine 20 mg/d	50	36	BDI (-)	54.0	Finland
					Citalopram 30 mg/day	49			54.0	
					Placebo	50			54.0	

Among the seven eligible articles, data from 992 participants who received antidepressants (mean age = 52.7 years, mean treatment duration = 9.5 weeks) and 684 participants who received a placebo (mean age = 52.5 years) were synthesized. All seven studies prohibited the use of any medications or hormone therapies thought to relieve menopausal symptoms or depression in the control groups. Of the seven studies, three recruited participants with a baseline definite diagnosis of MDD^11,12,29^, three excluded participants with baseline MDD^16,17,31^, and the other did not set any limitation regarding baseline MDD (i.e. included participants with the whole depressive spectrum)^29^. Regarding the antidepressants that were investigated in each RCT, most studies investigated only one antidepressant, including desvenlafaxine in three^11,16,30^, fluoxetine in one^12^, and paroxetine in one^28^. In addition, in the study by Cheng (2013), the authors included two antidepressant-treated groups with different dosages of desvenlafaxine^16^. The other two studies investigated two antidepressants at the same time, one with citalopram and fluoxetine^14^ and the other one with citalopram and venlafaxine^29^.

### Methodological quality of the included studies

Overall, we found that 65.3% (32/49 items), 22.5% (11/49 items), and 12.2% (6/49 items) of the included studies had a low, unclear, and high risk of bias, respectively. Unclear reporting of the allocation procedure or attrition bias of the studies further contributed to the risk of bias (Supplementary Table S4).

### Primary outcome

**Figure 2:**
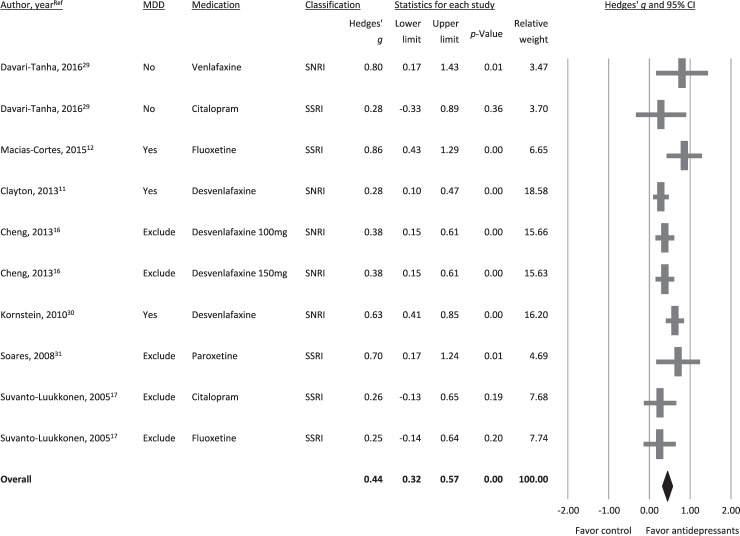
Forest plot of changes in depressive symptoms in menopausal women with antidepressant treatments compared to those without. Note significantly better improvements in severity of depressive symptoms (k = 10, Hedges’ g = 0.44, 95% CI = 0.32 to 0.57, *p* < 0.001) in subjects receiving antidepressants compared to that in controls.

The seven eligible studies included ten antidepressant-treated groups evaluating changes in the severity of depressive symptoms^11,12,16,17,29–31^, The meta-analysis showed that the participants randomized to receive antidepressants had a greater decrease in depressive symptoms relative to those who received a placebo (*k* = 7, Hedges’ *g* = 0.44, 95% CI = 0.32 to 0.57, *p* < 0.001) (Fig. 2) without significant heterogeneity (Q value = 13.73, df = 9, *I*^2^ = 34.43%, *p* = 0.13) or publication bias via Egger’s regression (t = 0.98, df = 8, *p* = 0.36). Sensitivity analysis where one study was excluded from analysis at a time revealed that no outlier among the included studies was biasing the overall ES estimates.

Few moderators could be tested in meta-regression analysis due to the limited number of independent datasets. Mean age (*p* = 0.06) and treatment duration (*p* = 0.10) did not appear to moderate the overall effect of antidepressants on depressive symptoms.

Subgroup analysis suggested that the participants randomized to receive either SSRIs (*k* = 4, Hedges’ *g* = 0.46, 95% CI = 0.20 to 0.73, *p* < 0.001) or SNRIs (*k* = 4, Hedges’ *g* = 0.43, 95% CI = 0.28 to 0.58, *p* < 0.001) had a significantly higher overall improvement in depressive symptoms relative to those receiving a placebo. Furthermore, the beneficial effects of antidepressants were also evident when only studies that excluded participants with MDD at baseline were considered (*k* = 3, Hedges’ *g* = 0.37, 95% CI = 0.24 to 0.51, *p* < 0.001).

### Secondary outcomes

#### Response rate

**Figure 3:**
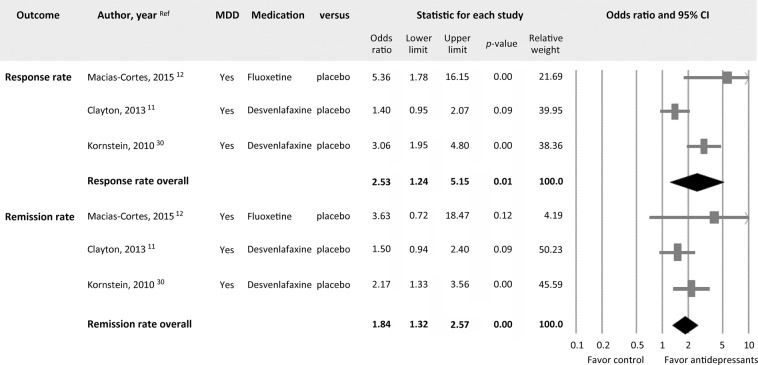
Forest plots of response and remission rates in menopausal women with antidepressant treatments compared to that in controls. Note significantly better response rate (k = 3, OR = 2.53, 95% CI = 1.24 to 5.15, *p* = 0.01) and remission rate (k = 3, OR = 1.84, 95% CI = 1.32 to 2.57, *p* < 0.001) in menopausal women with antidepressants than those in the controls.

**Figure 4:**
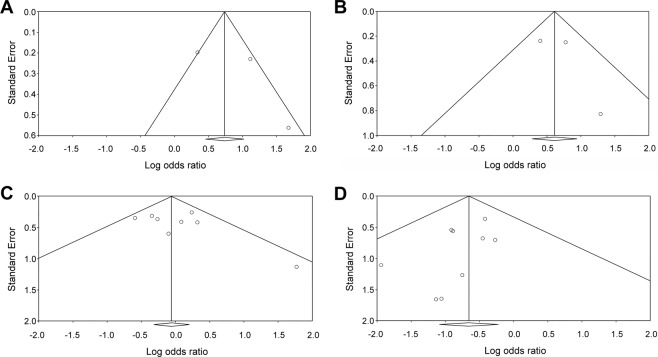
Funnel plots of meta-analysis on (**A**) response rate; (**B**) remission rate; (**C)** dropout rate, and (**D**) adverse event-related discontinuation rate.

When focusing on the three studies that provided data regarding response rates^11,12,30^, the current meta-analysis found that the participants randomized to receive antidepressants had a higher overall response rate than those who received a placebo (*k* = 3, OR = 2.53, 95% CI = 1.24 to 5.15, *p* = 0.01) (Fig. 3). There was, however, very large and significant heterogeneity (Q value = 9.58, df = 2, *I*^2^ = 79.12%, *p* = 0.01), and funnel plot inspection suggested publication bias (Fig. 4A). After adjusting for publication bias using Duval and Tweedie’s trim and fill procedure, the ES was rendered non-significant (adjusted OR = 1.40, 95% CI = 0.66 to 2.99).

#### Remission rate

Three included studies provided data regarding remission rates^11,12,30^. The meta-analysis showed that the participants receiving antidepressants had a higher remission rate than the controls (*k* = 3, OR = 1.84, 95% CI = 1.32 to 2.57, *p* < 0.001) (Fig. 3) without significant heterogeneity (Q value = 1.85, df = 2, *I*^2^ < 0.001%, *p* = 0.40) but significant publication bias via inspection of the funnel plot (Fig. 4B). The adjusted ESs using Duval and Tweedie’s trim and fill test remained statistically significant (adjusted OR = 1.50, 95% CI = 1.01 to 2.22).

#### Dropout rate

**Figure 5:**
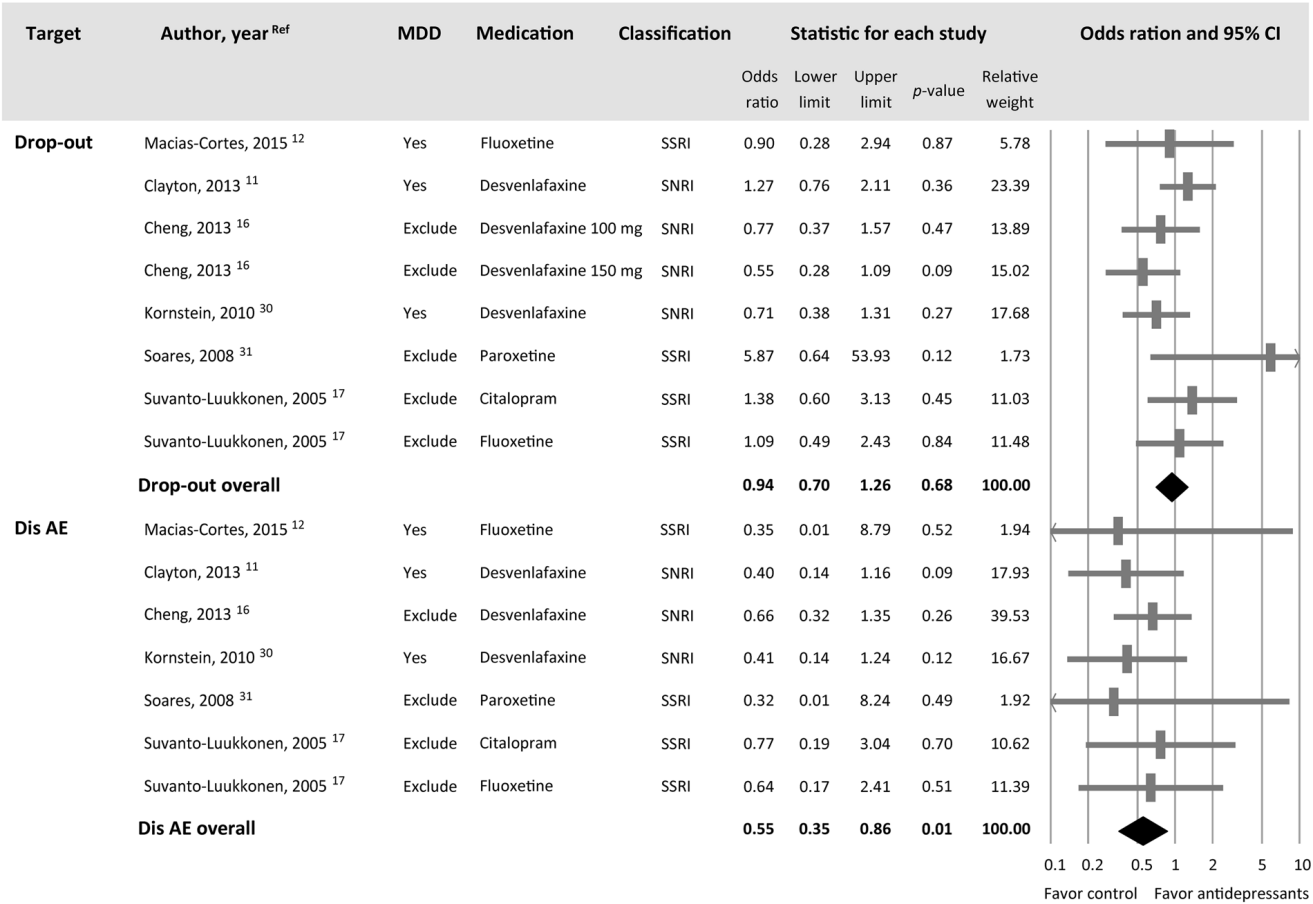
Forest plots of safety profile of antidepressants reflected by dropout rate and rate of study discontinuation due to adverse events in menopausal women with antidepressants and in those without. Note the non-significant difference in dropout rate between the two groups (k = 7, OR = 0.55, 95% CI = 0.35 to 0.86, *p* = 0.01) and significantly higher Dis AE rate in the subjects receiving antidepressants than those receiving placebos (k = 5, Hedges’ g = 0.25, 95% CI = 0.02 to 0.48, *p* = 0.03). Abbreviations: CI, confidence interval; Dis AE, study discontinuation due to adverse events; MA, meta-analysis; MDD, major depressive disorder; OR: odds ratio.

Six studies including eight antidepressant treatment groups provided data regarding dropout rates^11,12,16,17,30,31^. No significant differences in overall dropout rate were found between the participants receiving antidepressants relative to those receiving a placebo (*k* = 8, OR = 0.94, 95% CI = 0.70 to 1.26, *p* = 0.68) (Fig. 5). Furthermore, no significant heterogeneity (Q value = 8.33, df = 7, *I*^2^ < 16.0%, *p* = 0.30) was observed. However, inspection of the funnel plot suggested the presence of publication bias (Fig. 4C), although this ES remained non-significant after adjusting using Duval and Tweedie’s trim and fill procedure (adjusted OR = 0.91, 95% CI = 0.66 to 1.26). Of the seven studies having mentioned the dropout rates, five gave reasons for dropouts (Supplementary Table S3). For the treatment group, the reasons were adverse effects in four studies and ineffectiveness in another study. For the control groups, ineffectiveness was reported in three studies, while adverse effects and patient’s request were the reasons for dropouts in the other two studies, respectively.

In subgroup analysis, dropout rates were similar between the participants randomized to receive SSRIs compared to those receiving a placebo (*k* = 3, OR = 1.25, 95% CI = 0.76 to 2.07, *p* = 0.38). In addition, dropout rates were similar among the participants treated with SNRIs compared to those randomized to receive a placebo (*k* = 3, OR = 0.82, 95% CI = 0.57 to 1.19, *p* = 0.31).

#### Discontinuation rate due to adverse events

Six studies (seven antidepressant-treatment groups) provided data on discontinuation due to adverse events^11,12,16,17,30,31^. A significantly higher overall discontinuation rate due to adverse events was observed among the participants randomized to receive antidepressants relative to those receiving a placebo (*k* = 6, OR = 0.55, 95% CI = 0.35 to 0.86, *p* = 0.01) (Fig. 5) without significant heterogeneity (Q value = 1.30, df = 6, *I*^2^ < 0.001%, *p* = 0.97). The inspection of the funnel plot (Fig. 4D) suggested the possibility of publication bias. Nevertheless, the adjusted overall ES for this outcome remained statistically significant (adjusted OR = 0.56, 95% CI = 0.36 to 0.87).

In subgroup analysis, the discontinuation rate due to adverse events was similar between the participants who received SSRIs and those who received a placebo (*k* = 3, OR = 0.62, 95% CI = 0.26 to 1.51, *p* = 0.30). However, the discontinuation rate due to adverse events was higher in the participants who received SNRIs compared to those randomized to receive a placebo (*k* = 3, OR = 0.53, 95% CI = 0.31 to 0.89, *p* = 0.02). The side effects were mostly mild as assessed by the study investigators with the most common being nausea, dry mouth and headache in the antidepressant groups. Detailed information about the adverse events addressed in the included studies is summarized in Supplementary Table S3.

## Discussion

The results of the current meta-analysis were derived from seven RCTs including data from 1,676 participants, and suggested that antidepressant drug treatment, either with SSRIs or SNRIs, was efficacious for the management of depressive symptoms across the full spectrum of depressive disorders presenting during or after menopausal transition. Our results also suggested that antidepressant treatment during menopause was associated with higher response and remission rates compared to placebo. Furthermore, antidepressant treatment was not associated with higher dropout rates compared to placebo, although discontinuation rates due to adverse events were higher among the participants randomized to receive antidepressant compared to placebo. To the best of our knowledge, the current study is the first to try to synthesize evidence from RCTs on the efficacy, safety and tolerability of antidepressants for the treatment of depressive spectrum disorders during and after menopausal transition.

The present study had its strengths and limitations. Due the availability and inclusion of seven unique RCTs, we were able to perform a meta-analysis and investigate factors that may influence the therapeutic effects of antidepressants among peri- and post-menopausal women. Our results suggested that antidepressants could be efficacious for women who developed MDD during or after menopausal transition as well as for those presenting with subthreshold depressive symptoms. This is particularly relevant because cross-sectional and prospective studies have indicated that depressive symptoms in this population appear to occur on a *continuum* of severity^14,32,33^. Moreover, subthreshold depression may significantly impair the quality of life and functioning of this population^14^, which may also increase the risk of MDD in a subset of women during or after menopausal transition^32,33^. Furthermore, most of the studies seemed to have fair quality, while there was only dominant unclear risk in the item of allocation concealment. However, most of the studies were conducted in North America, with only one from the Middle East^29^ and one from North Europe^17^. Therefore, extrapolation of the results to other populations may not be justified. In addition, treatment with antidepressants was associated with a higher likelihood of achieving response and remission relative to a placebo. However, there was evidence of publication bias on the overall effects of antidepressants on treatment response, and hence the results should be interpreted with caution since this effect was rendered non-significant following adjustments for publication bias. It is also worth noting that only three trials provided data regarding response and remission rates.

Moreover, as in most meta-analyses, another limitation of the current study was the heterogeneity of the included studies in terms of study duration, drug dosage, the use of different depression scales and different versions (e.g., Hamilton depression rating scale). Therefore, we performed subgroup analysis and meta-regression to investigate how different factors may affect the study results. Meta-regression showed that certain factors such as duration and age did not affect our results, and our subgroup analysis demonstrated that antidepressant treatment was still effective for those suffering from subthreshold depression. Nevertheless, the number of studies was too small to allow other meaningful subgrouping or meta-regression analyses. Besides, there was heterogeneity in some estimates, the sources of which were explored through subgroup and meta-regression analyses. However, due to the relatively small number of studies, the results from these analyses should be regarded as exploratory instead of conclusive. Furthermore, in recent years, perimenopausal depression is considered a distinct subtype of depression that warrants a unique rating scale for evaluation^3^. Nevertheless, most studies in the present meta-analysis were old and did not use criteria specified for perimenopausal depression^3^. The issue was further complicated by the fact that most studies included a mixed population of women during menopausal transition and in the postmenopausal phase. Therefore, whether the instruments reported in those studies could capture the complex symptoms of perimenopausal depression remains to be elucidated. Finally, the overall body of evidence remains limited in this area.

Because the treatment of depression during menopause remains a clinical challenge, the findings of the present study had its clinical implications. The current meta-analysis indicates that antidepressants could be efficacious for the treatment of this condition in this vulnerability period of the female reproductive cycle. However, only three RCTs included participants with a definite diagnosis of MDD^11,12,30^. An evidence-based psychotherapeutic approach for depression (e.g., cognitive behavioral therapy or interpersonal therapy) could initially be offered to menopausal women with subthreshold depression, although the evidence base for this practice has mostly been extrapolated from psychotherapeutic trials that have been conducted in general cases of depression^14^. Interestingly, we did not identify any RCTs on the effects of tricyclic antidepressant agents in postmenopausal women with depression. Therefore, this meta-analysis provides evidence for the use of SSRIs and SNRIs as treatments for depression in this population. Specifically, the antidepressants fluoxetine^12,17^, citalopram^17,29^, paroxetine^31^, desvenlafaxine^11,16,30^ and venlafaxine^29^ were tested across the included RCTs. Further research is warranted to investigate the effects of other antidepressants in this population.

There was no significant difference in dropout rate between the participants treated with antidepressants and those who received a placebo. However, antidepressant treatment was associated with a greater likelihood of discontinuation due to adverse events. This is consistent with an increasing number of studies that have raised concerns regarding the safety and tolerability of newer generation antidepressants, including SSRIs and SNRIs^34^. Such concerns should be weighed when considering the use of antidepressants, especially for women during or after menopausal transition with less severe forms of depression^35^.

The current systematic review and meta-analysis may provide new directions for research. First, only acute antidepressant trials were identified. However, depressive disorders in postmenopausal women appear to have heterogenous symptom trajectories^32,33^, and further investigations are warranted to investigate the benefits of maintenance treatment with antidepressants for depressive disorders in this population (i.e., the design of long-term RCTs). In addition, our subgroup analysis suggested that SNRIs but not SSRIs were associated with higher discontinuation rates due to adverse events relative to a placebo. However, further RCTs are needed to confirm this finding. It has been suggested that the presence of vasomotor symptoms during menopause may contribute to the development and persistence of depressive disorders during this phase of the female reproductive cycle^14^, and also that low-dose paroxetine and SNRIs could improve these symptoms^36^. Therefore, further research is needed to investigate whether the amelioration of vasomotor disturbances could contribute to the beneficial effects of antidepressants seen in the current study. Finally, the evidence base regarding options for the treatment of depressive disorders during menopause remains limited. The design of new RCTs is a necessary step before firm conclusions regarding the comparative efficacy and tolerability of various pharmacological treatments can be made.

## Conclusions

The current systematic review and meta-analysis provides evidence that antidepressants are effective for the treatment of depressive disorders for women during and after menopausal transition. Long-term RCTs are required to investigate the efficacy, safety, and tolerability of maintenance treatment with antidepressants during menopause.

## Supplementary information

Supplementary information.

## Data Availability

Yu-Shian Cheng (Y.-S.C.) and Ping-Tao Tseng (Y.-S.C.) both had full access to all the data in this study, conducted the data analysis, and took responsibility for integrity of the data and accuracy of the data analysis. The data of the current study are available within the article and its supplementary materials. For further information, requests shall be directed to the corresponding author.
