# Author Correction: Antidepressants during and after Menopausal Transition: A Systematic Review and Meta-Analysis

**DOI:** 10.1038/s41598-022-18882-6

**Published:** 2022-08-30

**Authors:** Ching-Kuan Wu, Ping-Tao Tseng, Ming-Kung Wu, Dian-Jeng Li, Tien-Yu Chen, Fu-Chen Kuo, Brendon Stubbs, Andre F. Carvalho, Yen-Wen Chen, Pao-Yen Lin, Yu-Shian Cheng, Cheuk-Kwan Sun

**Affiliations:** 1 Department of Psychiatry, Tsyr-Huey Mental Hospital, Kaohsiung Jen-Ai’s Home, Kaohsiung, Taiwan; 2 WinShine Clinics in Specialty of Psychiatry, Kaohsiung City, Taiwan; 3 Department of Psychiatry, Kaohsiung Chang Gung Memorial Hospital and Chang Gung University College of Medicine, Kaohsiung, Taiwan; 4 Graduate Institute of Medicine, College of Medicine, Kaohsiung Medical University, Kaohsiung, Taiwan; 5 Department of Addiction Science, Kaohsiung Municipal Kai-Syuan Psychiatric Hospital, Kaohsiung City, Taiwan; 6 Department of Psychiatry, Tri-Service General Hospital, School of Medicine, National Defense Medical Center, Taipei, Taiwan; 7 Institute of Brain Science, National Yang-Ming University, Taipei, Taiwan; 8 Department of Obstetrics & Gynecology, E-Da Hospital, Kaohsiung, Taiwan; 9 School of Medicine, College of Medicine, I-Shou University, Kaohsiung, Taiwan; 10 Physiotherapy Department, South London and Maudsley NHS Foundation Trust, London, UK; 11 Department of Psychological Medicine, Institute of Psychiatry, Psychology and Neuroscience (IoPPN), King’s College London, De Crespigny Park, London, UK; 12 Faculty of Health, Social Care and Education, Anglia Ruskin University, Chelmsford, UK; 13 Department of Psychiatry, University of Toronto, Toronto, ON Canada; 14 Centre for Addiction & Mental Health (CAMH), Toronto, ON Canada; 15 Prospect Clinic for Otorhinolaryngology & Neurology, Kaohsiung, Taiwan; 16 Institute for Translational Research in Biomedical Sciences, Kaohsiung Chang Gung Memorial Hospital, Kaohsiung, Taiwan; 17 Department of Emergency Medicine, E-Da Hospital, Kaohsiung, Taiwan; 18 Institute of Biomedical Sciences, National Sun Yat-Sen University, Kaohsiung, Taiwan; 19 Department of Chemical Engineering and Institute of Biotechnology and Chemical Engineering, I-Shou University, Kaohsiung, Taiwan; 20 Institute of Medical Science and Technology, National Sun Yatsen University, Kaohsiung, Taiwan

Correction to: *Scientific Reports* https://doi.org/10.1038/s41598-020-64910-8, published online 15 May 2020

The original version of this Article contained errors.

In Figure 2, the data corresponding to the low-dose Desvenlafaxine (100 mg/day) and high-dose Desvenlafaxine (150 mg/day) treatment arms of Cheng, 2013 were not labelled. The original Figure 2 and accompanying legend appear below.

**Figure 2:**
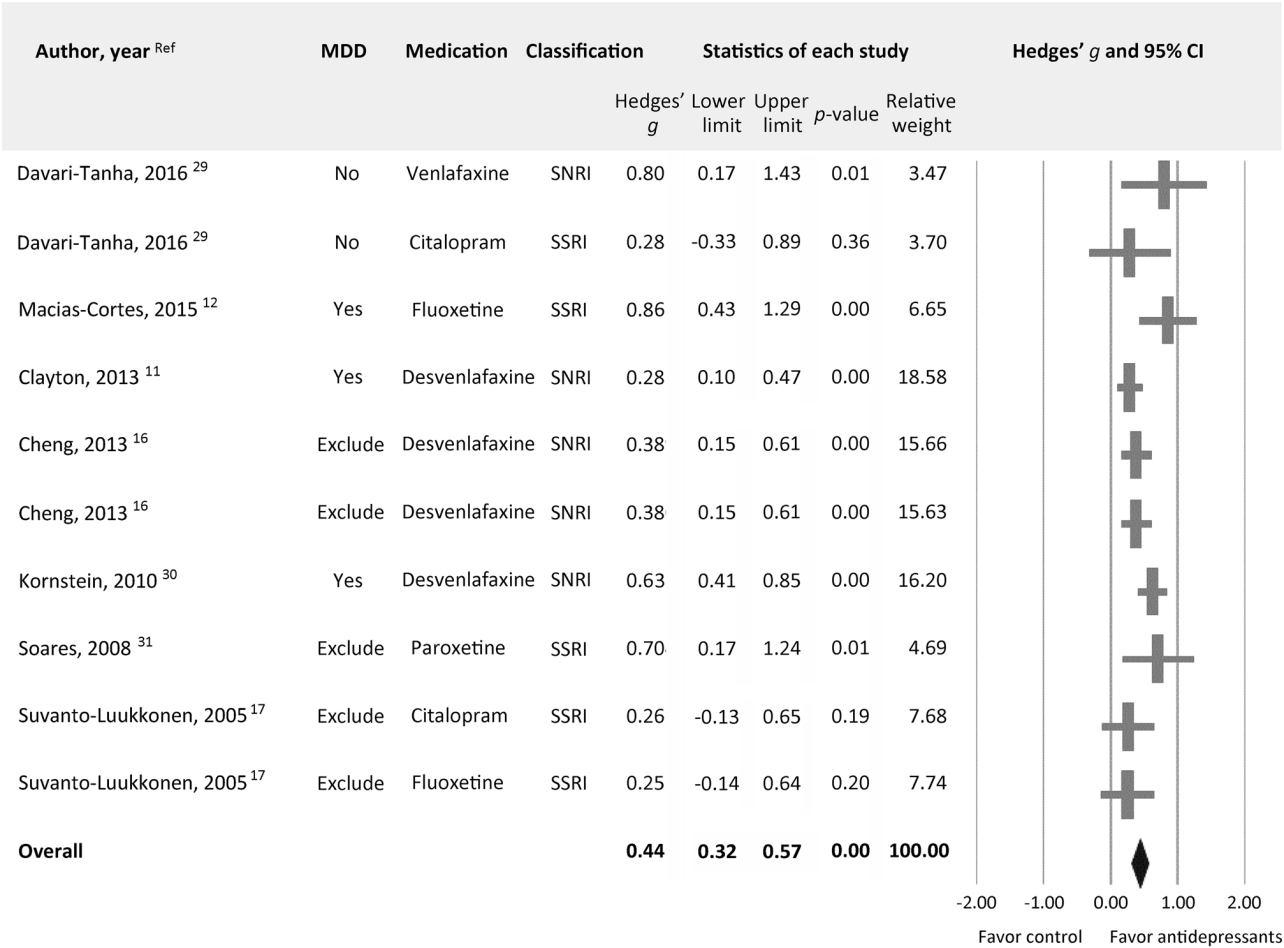
Forest plot of changes in depressive symptoms in menopausal women with antidepressant treatments compared to those without. Note significantly better improvements in severity of depressive symptoms (k = 10, Hedges’ g = 0.44, 95% CI 0.32 to 0.57, *p* < 0.001) in subjects receiving antidepressants compared to that in controls.

Additionally, in the Methods section, under the sub-heading ‘Guidelines and protocol’,

“This systematic review and meta-analysis was conducted according to the guidelines presented in the *Preferred Reporting Items for Systematic Reviews and Meta-Analyses* (PRISMA) statement^18^ (Supplementary Table S1). An a priori defined but unpublished protocol (available upon request to the authors) that was approved by the Institutional Review Board of Tri-Service General Hospital (TSGHIRB: B-105-12) was followed.”

now reads:

“This systematic review and meta-analysis was conducted according to the guidelines presented in the *Preferred Reporting Items for Systematic Reviews and Meta-Analyses* (PRISMA) statement^18^ (Supplementary Table S1).”

The study protocol is reported in detail in the Methods section of the Article.

The original Article has been corrected.

