# Supplementary material for: Antidepressants during and after Menopausal Transition: A Systematic Review and Meta-Analysis
Source: Sci Rep. 2020 May 15;10:8026. doi: 10.1038/s41598-020-64910-8 (PMC7228969; doi:10.1038/s41598-020-64910-8)
Supplement: Supplementary file 1 — Supplementary information. [file 41598_2020_64910_MOESM1_ESM.doc]

**Antidepressants during and after Menopausal Transition: A Systematic Review and Meta-Analysis**

**Running head:** Antidepressants for menopausal depression

**Keywords***:* menopause; antidepressants; meta-analysis; efficacy; tolerability

Ching-Kuan Wu, MD ^1,†^; Ping-Tao Tseng, MD ^2,†^; Ming-Kung Wu, MD ^3,†^; Dian-Jeng Li, MD ^4,5^; Tien-Yu Chen, MD ^6,7^; Fu-Chen Kuo, MD ^8,9^; Brendon Stubbs, MD ^10,11,12^; Andre F Carvalho, MD ^13,14^; Yen-Wen Chen, MD ^15^; Pao-Yen Lin, MD ^3,16^; Yu-Shian Cheng, MD ^1^,*; Cheuk-Kwan Sun, MD, PhD ^9,17^,*

^1^ Department of Psychiatry, Tsyr-Huey Mental Hospital, Kaohsiung Jen-Ai’s Home, Taiwan

^2^ WinShine Clinics in Specialty of Psychiatry, Kaohsiung City, Taiwan

^3^ Department of Psychiatry, Kaohsiung Chang Gung Memorial Hospital and Chang Gung University College of Medicine, Kaohsiung, Taiwan

^4^ Graduate Institute of Medicine, College of Medicine, Kaohsiung Medical University, Taiwan

^5^ Department of Addiction Science, Kaohsiung Municipal Kai-Syuan Psychiatric Hospital, Kaohsiung City, Taiwan

^6^ Department of Psychiatry, Tri-Service General Hospital; School of Medicine, National Defense Medical Center, Taipei, Taiwan

^7^ Institute of Brain Science, National Yang-Ming University, Taipei, Taiwan

^8^ Department of Obstetrics & Gynecology, E-Da Hospital, Kaohsiung, Taiwan

^9^ School of Medicine, College of Medicine, I-Shou University, Kaohsiung, Taiwan

^10^ Physiotherapy Department, South London and Maudsley NHS Foundation Trust, London, UK

^11^ Department of Psychological Medicine, Institute of Psychiatry, Psychology and Neuroscience (IoPPN), King's College London, De Crespigny Park, London, UK

^12^ Faculty of Health, Social Care and Education, Anglia Ruskin University, Chelmsford, UK

^13^ Department of Psychiatry, University of Toronto, Toronto, ON, Canada.

^14^ Centre for Addiction & Mental Health (CAMH), Toronto, ON, Canada

^15^ Prospect Clinic for Otorhinolaryngology & Neurology

^16^ Institute for Translational Research in Biomedical Sciences, Kaohsiung Chang Gung Memorial Hospital, Kaohsiung, Taiwan

^17^ Department of Medical Research E-Da Hospital, Kaohsiung, Taiwan

^†^contributed equally as the first author

*contributed equally as the corresponding author

**Please address correspondence to:**

Cheuk-Kwan Sun, MD, PhD

Affiliation: Department of Medical Research, E-Da Hospital, I-Shou University School of Medicine for International Students, Kaohsiung, Taiwan

Address: No.1, Yida Road, Jiaosu Village, Yanchao District, Kaohsiung City 82445, Taiwan

Telephone: 886-7-6150011 ext. 1007

Fax: 886-7-615-0945

Email: [lawrence.c.k.sun@gmail.com](mailto:lawrence.c.k.sun@gmail.com); [ed105983@edah.org.tw](mailto:ed105983@edah.org.tw)

OR

Yu-Shian Cheng, MD

Affiliation: Department of Psychiatry, Tsyr-Huey Mental Hospital, Kaohsiung Jen-Ai’s Home, Taiwan

Address: No. 509, Fengping 1st Rd., Daliao Dist., Kaohsiung City 831, Taiwan

Telephone: 886-7-7030315

Fax: 886-7-7012624

Email: [n043283@gmail.com](mailto:n043283@gmail.com)

**Supplementary Table S1.** PRISMA checklist of current meta-analysis^1^

| **Section/Topic** | **#** | **Checklist Item** | **Reported on Page #** |
| --- | --- | --- | --- |
| **TITLE** | | | |
| Title | 1 | Identify the report as a systematic review, meta-analysis, or both.  Antidepressants during Menopausal Transition: A Systematic Review and Meta-Analysis | 1 |
| **ABSTRACT** | | | |
| Structured summary | 2 | Provide a structured summary including, as applicable: background; objectives; data sources; study eligibility criteria, participants, and interventions; study appraisal and synthesis methods; results; limitations; conclusions and implications of key findings; systematic review registration number.  Seven trials involving 1,676 participants showed significant improvement in depressive symptoms [k = 7, Hedges’ g = 0.44, 95% confidence interval (CI) = 0.32 to 0.57, *p* < 0.001] relative to that in controls. Furthermore, response [k = 3, odds ratio (OR) = 2.53, 95% CI = 1.24 to 5.15, p = 0.01] and remission (k = 3, OR = 1.84, 95% CI = 1.32 to 2.57, *p* < 0.001) rates were significantly higher in antidepressant-treated groups compared to those with placebos. Although drop-out rates did not differ between antidepressant and placebo groups (k = 6, OR = 0.93, 95% CI = 0.70 to 1.26, *p* = 0.68), the rate of discontinuation due to adverse events was significantly higher in antidepressant-treated groups (k = 6, OR = 0.55, 95% CI = 0.35 to 0.86, *p* = 0.01). Subgroup analysis indicated that antidepressants were also efficacious for depressive symptoms in those without diagnosis of MDD. | 3-4 |
| **INTRODUCTION** | | | |
| Rationale | 3 | Describe the rationale for the review in the context of what is already known.  Accumulating evidence indicates that women appear to be at a particularly higher risk of the emergence of major depressive disorder (MDD) and also depressive symptoms not severe enough to meet the diagnostic criteria of MDD during menopausal transition. The management of depressive symptoms among peri- and post-menopausal women can be challenging. | 5 |
| Objectives | 4 | Provide an explicit statement of questions being addressed with reference to participants, interventions, comparisons, outcomes, and study design (PICOS).  The current study aimed at providing a comprehensive systematic review and meta-analysis of all randomized controlled clinical trials (RCTs) evaluating the effects of antidepressants in peri- and post-menopausal women with the whole spectrum of depressive disorders during menopausal transition. In addition, we aimed at: (1) assessing the therapeutic effects of antidepressant treatment in this population; (2) evaluating whether potential benefits of antidepressant agents differ in those with full-blown MDD compared to those experiencing subthreshold depression; and (3) investigating the safety and tolerability of antidepressants. | 5-6 |
| **METHODS** | | | |
| Protocol and registration | 5 | Indicate if a review protocol exists, if and where it can be accessed (e.g., Web address), and, if available, provide registration information including registration number.  This systematic review and meta-analysis was conducted according to the guidelines presented in the *Preferred Reporting Items for Systematic Reviews and Meta-Analyses* (PRISMA) statement. | 7-8 |
| Eligibility criteria | 6 | Specify study characteristics (e.g., PICOS, length of follow-up) and report characteristics (e.g., years considered, language, publication status) used as criteria for eligibility, giving rationale.  (1) Peer-reviewed articles investigating the efficacy of antidepressants against depressive symptoms in menopausal women meeting the criteria for MDD or experiencing subthreshold depressive symptoms; and (2) articles that were controlled trials conducted in humans. No language restrictions were applied. | 7-8 |
| Information sources | 7 | Describe all information sources (e.g., databases with dates of coverage, contact with study authors to identify additional studies) in the search and date last searched.  PubMed/MEDLINE, Cochrane Library, EMBASE and Science Direct electronic databases from inception to February 1, 2020. | 8-9 |
| Search | 8 | Present full electronic search strategy for at least one database, including any limits used, such that it could be repeated.  Systematic literature search was performed using the following keywords: (antidepressants and [perimenopause or postmenopause or menopause] and [depression or depressive disorder or mood]). In addition, the ClinicalTrials.gov database was searched using the following search string: ([antidepressant] AND [depression and menopause]). | 8-9 |
| Study selection | 9 | State the process for selecting studies (i.e., screening, eligibility, included in systematic review, and, if applicable, included in the meta-analysis).  The inclusion criteria were: (1) peer-reviewed articles investigating the efficacy of antidepressants on depressive symptoms in menopausal women meeting the criteria for MDD or experiencing subthreshold depressive symptoms; and (2) articles that were controlled trials conducted in humans. No language restrictions were applied. | 8-9 |
| Data collection process | 10 | Describe method of data extraction from reports (e.g., piloted forms, independently, in duplicate) and any processes for obtaining and confirming data from investigators.  Two independent authors extracted data from the eligible studies into a database of pre-determined variables of interest. | 8-9 |
| Data items | 11 | List and define all variables for which data were sought (e.g., PICOS, funding sources) and any assumptions and simplifications made.  Variables included mean age (years), mean body mass index (BMI), duration of antidepressant treatment (weeks), and ethnicity (Caucasian, African American, Hispanic, or Asian). The corresponding authors of the retrieved studies were contacted by emails to request additional data on at least two different occasions 1 week apart whenever variables of interest were not available. | 8-9 |
| Risk of bias in individual studies | 12 | Describe methods used for assessing risk of bias of individual studies (including specification of whether this was done at the study or outcome level), and how this information is to be used in any data synthesis.  Two independent authors (YS Cheng and PT Tseng) evaluated the risk of bias (inter-rater reliability, 0.85) for each domain described in the Cochrane risk of bias tool. | 9-10 |
| Summary measures | 13 | State the principal summary measures (e.g., risk ratio, difference in means). | 9-10 |
| Synthesis of results | 14 | Describe the methods of handling data and combining results of studies, if done, including measures of consistency (e.g., I^2^) for each meta-analysis.  Changes in effect sizes (ESs) of depressive symptoms between groups were analyzed using Hedges’ *g* and 95% confidence intervals (95% CIs). We calculated odds ratios (ORs) with 95% CIs for secondary outcomes using dichotomous items (such as response and remission rates). | 9-11 |
| Risk of bias across studies | 15 | Specify any assessment of risk of bias that may affect the cumulative evidence (e.g., publication bias, selective reporting within studies).  With the automated program of Comprehensive Meta-Analysis software version 3, we arranged sensitivity analysis to verify whether an outlier could be biasing our ES estimates. To be specific, to comprehensively evaluate the potential bias contributed to an outlier, we removed one study at a time from the analysis and completed the examination of potential bias contributed by each study included in the current study. | 9-11 |
| Additional analyses | 16 | Describe methods of additional analyses (e.g., sensitivity or subgroup analyses, meta-regression), if done, indicating which were pre-specified.  To evaluate potential sources of heterogeneity and confounding effects, we performed meta-regression and subgroup meta-analyses. | 9-11 |
| **RESULTS** | | | |
| Study selection | 17 | Give numbers of studies screened, assessed for eligibility, and included in the review, with reasons for exclusions at each stage, ideally with a flow diagram.  After excluding duplicates, 42 full-text articles were assessed for eligibility. Among them, 35 were excluded due to the following reasons including: (1) No placebo (n=27), (2) Duplicate sample source (n=3), (3) Inclusion of participants not around time of menopause (n=1), (4) Lack of adequate outcome measurement or baseline data for analysis (n=3), and (5) Combination therapy, not just antidepressant (n=1) [Supplementary Table S2 (available online)]. Therefore, seven articles were eligible for the current meta-analysis (Table 1) | 12-13 |
| Study characteristics | 18 | For each study, present characteristics for which data were extracted (e.g., study size, PICOS, follow-up period) and provide the citations.  Among the seven eligible articles, data from 992 participants who received antidepressants (mean age = 52.7 years, mean treatment duration = 9.5 weeks) and 684 participants who received a placebo (mean age = 52.5 years) were synthesized. | 12-13 table 1 |
| Risk of bias within studies | 19 | Present data on risk of bias of each study and, if available, any outcome level assessment (see item 12).  Overall, we found that 65.3% (32/49 items), 22.5% (11/49 items), and 12.2% (6/49 items) of the included studies had a low, unclear, and high risk of bias, respectively. Unclear reporting of the allocation procedure or attrition bias of the studies further contributed to the risk of bias (Supplementary Table S4). | 12-14 |
| Results of individual studies | 20 | For all outcomes considered (benefits or harms), present, for each study: (a) simple summary data for each intervention group (b) effect estimates and confidence intervals, ideally with a forest plot.  Please see the forest plot. | 12-17, table 1, figure 2 |
| Synthesis of results | 21 | Present the main results of the review. If meta-analyses done, include for each, confidence intervals and measures of consistency.  Please see the forest plot. | 12-17, figure 2 |
| Risk of bias across studies | 22 | Present results of any assessment of risk of bias across studies (see Item 15).  Please see the funnel plot. | 13-16 |
| Additional analysis | 23 | Give results of additional analyses, if done (e.g., sensitivity or subgroup analyses, meta-regression [see Item 16]).  Subgroup analysis suggested that the participants randomized to receive either SSRIs (*k* = 4, Hedges’ *g* = 0.46, 95% CI = 0.20 to 0.73, *p* < 0.001) or SNRIs (*k* = 4, Hedges’ *g* = 0.43, 95% CI = 0.28 to 0.58, *p* < 0.001) had a significantly higher overall improvement in depressive symptoms relative to those receiving a placebo. Furthermore, the beneficial effects of antidepressants were also evident when only studies that excluded participants with MDD at baseline were considered (*k* = 3, Hedges’ *g* = 0.37, 95% CI = 0.24 to 0.51, *p* < 0.001). | 12-17, figure 3 |
| **DISCUSSION** | | | |
| Summary of evidence | 24 | Summarize the main findings including the strength of evidence for each main outcome; consider their relevance to key groups (e.g., healthcare providers, users, and policy makers).  The results of the current meta-analysis were derived from seven RCTs including data from 1,676 participants, and suggested that antidepressant treatment, either with SSRIs or SNRIs, was efficacious for the management of depressive symptoms across the full spectrum of depressive disorders presenting during and after menopausal transition. Our results also suggested that antidepressant treatment during menopause was associated with higher response and remission rates compared to placebo. Furthermore, antidepressant treatment was not associated with higher drop-out rates compared to placebo, although discontinuation rates due to adverse events were higher among the participants randomized to receive antidepressant compared to placebo. | 18-22 |
| Limitations | 25 | Discuss limitations at study and outcome level (e.g., risk of bias), and at review-level (e.g., incomplete retrieval of identified research, reporting bias).  As in most meta-analyses, a limitation of the current study was the heterogeneity of the included studies in terms of study duration, drug dosage, the use of different depression scales and different versions (e.g., Hamilton depression rating scale). Although subgroup analysis would have shed light on the effects of different factors (e.g., duration and dosage) on the results, the number of studies was too small to allow meaningful subgrouping. | 18-19 |
| Conclusions | 26 | Provide a general interpretation of the results in the context of other evidence, and implications for future research.  The current systematic review and meta-analysis provides evidence that antidepressants are effective for the treatment of depressive disorders during menopause. Long-term RCTs are required to investigate the efficacy, safety, and tolerability of maintenance treatment with antidepressants during menopause. | 22 |
| **FUNDING** | | | |
| Funding | 27 | Describe sources of funding for the systematic review and other support (e.g., supply of data); role of funders for the systematic review.  The authors of this work did not receive any grants or financial support and reported no financial interests or potential conflicts of interest in this study. | 22 |

**Reference**

1. Moher, D., Liberati, A., [Tetzlaff, J](https://www.ncbi.nlm.nih.gov/pubmed/?term=Tetzlaff J%5BAuthor%5D&cauthor=true&cauthor_uid=19621072)., [Altman, D. G](https://www.ncbi.nlm.nih.gov/pubmed/?term=Altman DG%5BAuthor%5D&cauthor=true&cauthor_uid=19621072). & PRISMA Group. Preferred reporting items for systematic reviews and meta-analyses: the PRISMA statement. PLoS Med **6**, e1000097, https://doi.org/10.1371/journal.pmed.1000097 (2009).

**Supplementary Table S2.** Reasons for exclusion

1. No placebo (n=27)

1 Iglesias Garcia C, Ocio Leon S, Ortigosa Digon JC, Merino Garcia MJ, Alonso Villa MJ, Fernandez Palicio L, Alonso Rionda JL, Garcia Fernandez F, Rodriguez Colubi L, Toimil Iglesias A: Comparison of the effectiveness of venlafaxine in peri- and postmenopausal patients with major depressive disorder. Actas espanolas de psiquiatria 2010;38:326-331.

2 Huang YX, Song L, Zhang X, Lun WW, Pan C, Huang YS: [Clinical study of combined treatment of remifemin and paroxetine for perimenopausal depression]. Zhonghua yi xue za zhi 2013;93:600-602.

3 Iglesias C, Pato E, Ocio S, Ortigosa JC, Santamarina S, Merino MJ, Alonso MJ, Fernandez L, Alonso JL, Rodriguez L: [Treatment with venlafaxine extended release for climacteric women with depression or anxiety diagnosis. An open-label study]. Actas espanolas de psiquiatria 2009;37:137-142.

4 Ushiroyama T, Ikeda A, Ueki M: Evaluation of double-blind comparison of fluvoxamine and paroxetine in the treatment of depressed outpatients in menopause transition. Journal of medicine 2004;35:151-162.

5 Pae CU, Mandelli L, Kim TS, Han C, Masand PS, Marks DM, Patkar AA, Steffens DC, De Ronchi D, Serretti A: Effectiveness of antidepressant treatments in pre-menopausal versus post-menopausal women: a pilot study on differential effects of sex hormones on antidepressant effects. Biomedicine & pharmacotherapy = Biomedecine & pharmacotherapie 2009;63:228-235.

6 Zanardi R, Rossini D, Magri L, Malaguti A, Colombo C, Smeraldi E: Response to SSRIs and role of the hormonal therapy in post-menopausal depression. European neuropsychopharmacology : the journal of the European College of Neuropsychopharmacology 2007;17:400-405.

7 Freeman MP, Hill R, Brumbach BH: Escitalopram for perimenopausal depression: an open-label pilot study. J Womens Health (Larchmt) 2006;15:857-861.

8 Rasgon NL, Dunkin J, Fairbanks L, Altshuler LL, Troung C, Elman S, Wroolie TE, Brunhuber MV, Rapkin A: Estrogen and response to sertraline in postmenopausal women with major depressive disorder: a pilot study. J Psychiatr Res 2007;41:338-343.

9 Dias RS, Kerr-Correa F, Moreno RA, Trinca LA, Pontes A, Halbe HW, Gianfaldoni A, Dalben IS: Efficacy of hormone therapy with and without methyltestosterone augmentation of venlafaxine in the treatment of postmenopausal depression: a double-blind controlled pilot study. Menopause 2006;13:202-211.

10 Ladd CO, Newport DJ, Ragan KA, Loughhead A, Stowe ZN: Venlafaxine in the treatment of depressive and vasomotor symptoms in women with perimenopausal depression. Depress Anxiety 2005;22:94-97.

11 Morgan ML, Cook IA, Rapkin AJ, Leuchter AF: Estrogen augmentation of antidepressants in perimenopausal depression: a pilot study. The Journal of clinical psychiatry 2005;66:774-780.

12 Berlanga C, Mendieta D, Alva G, del Carmen Lara M: Failure of tibolone to potentiate the pharmacological effect of fluoxetine in postmenopausal major depression. J Womens Health (Larchmt) 2003;12:33-39.

13 Chaby L, Grinsztein A, Weitzman JJ, de Bodinat C, Dagens V: [Anxiety-related and depressive disorders in women during the premenopausal and menopausal period. Study of the efficacy and acceptability of tianeptine versus maprotiline]. Presse medicale (Paris, France : 1983) 1993;22:1133-1138.

14 Pae CU, Mandelli L, Han C, Ham BJ, Masand PS, Patkar AA, Steffens DC, De Ronchi D, Serretti A: Do estradiol levels influence on the cognitive function during antidepressant treatments in post-menopausal women with major depressive disorder? A comparison with pre-menopausal women. Neuro endocrinology letters 2008;29:500-506.

15 Vermeiden M, van den Broek WW, Mulder PG, Birkenhager TK: Influence of gender and menopausal status on antidepressant treatment response in depressed inpatients. J Psychopharmacol 2010;24:497-502.

16 Joffe H, Groninger H, Soares CN, Nonacs R, Cohen LS: An open trial of mirtazapine in menopausal women with depression unresponsive to estrogen replacement therapy. Journal of women's health & gender-based medicine 2001;10:999-1004.

17 Chojnacki C, Walecka-Kapica E, Klupinska G, Pawlowicz M, Blonska A, Chojnacki J: Effects of fluoxetine and melatonin on mood, sleep quality and body mass index in postmenopausal women. Journal of physiology and pharmacology : an official journal of the Polish Physiological Society 2015;66:665-671.

18 Barton DL, Loprinzi CL, Novotny P, Shanafelt T, Sloan J, Wahner-Roedler D, Rummans TA, Christensen B, Dakhill SR, Martin LS: Pilot evaluation of citalopram for the relief of hot flashes. The journal of supportive oncology 2003;1:47-51.

19 Soares CN, Thase ME, Clayton A, Guico-Pabia CJ, Focht K, Jiang Q, Kornstein SG, Ninan P, Kane CP, Cohen LS: Desvenlafaxine and escitalopram for the treatment of postmenopausal women with major depressive disorder. Menopause 2010;17:700-711.

20 Freeman MP, Hirschberg AM, Wang B, Petrillo LF, Connors S, Regan S, Joffe H, Cohen LS: Duloxetine for major depressive disorder and daytime and nighttime hot flashes associated with the menopausal transition. Maturitas 2013;75:170-174.

21 Soares CN, Thase ME, Clayton A, Guico-Pabia CJ, Focht K, Jiang Q, Kornstein SG, Ninan PT, Kane CP: Open-label treatment with desvenlafaxine in postmenopausal women with major depressive disorder not responding to acute treatment with desvenlafaxine or escitalopram. CNS Drugs 2011;25:227-238.

22 Defronzo Dobkin R, Menza M, Allen LA, Marin H, Bienfait KL, Tiu J, Howarth J: Escitalopram reduces hot flashes in nondepressed menopausal women: A pilot study. Annals of clinical psychiatry : official journal of the American Academy of Clinical Psychiatrists 2009;21:70-76.

23 Joffe H, Soares CN, Petrillo LF, Viguera AC, Somley BL, Koch JK, Cohen LS: Treatment of depression and menopause-related symptoms with the serotonin-norepinephrine reuptake inhibitor duloxetine. The Journal of clinical psychiatry 2007;68:943-950.

24 Westlund Tam L, Parry BL: Does estrogen enhance the antidepressant effects of fluoxetine? J Affect Disord 2003;77:87-92.

25 Oktem M, Eroglu D, Karahan HB, Taskintuna N, Kuscu E, Zeyneloglu HB: Black cohosh and fluoxetine in the treatment of postmenopausal symptoms: a prospective, randomized trial. Adv Ther 2007;24:448-461.

26 Soares CN, Arsenio H, Joffe H, Bankier B, Cassano P, Petrillo LF, Cohen LS: Escitalopram versus ethinyl estradiol and norethindrone acetate for symptomatic peri- and postmenopausal women: impact on depression, vasomotor symptoms, sleep, and quality of life. Menopause 2006;13:780-786.

27 Yasui T, Yamada M, Uemura H, Ueno S, Numata S, Ohmori T, Tsuchiya N, Noguchi M, Yuzurihara M, Kase Y, Irahara M: Changes in circulating cytokine levels in midlife women with psychological symptoms with selective serotonin reuptake inhibitor and Japanese traditional medicine. Maturitas 2009;62:146-152.

2. Duplicate sample source (n=3)

28 Macias-Cortes Edel C, Aguilar-Faisal L, Asbun-Bojalil J: Efficacy of individualized homeopathic treatment and fluoxetine for moderate to severe depression in peri- and postmenopausal women (HOMDEP-MENOP): study protocol for a randomized, double-dummy, double-blind, placebo-controlled trial. Trials 2013;14:105.

29 Kornstein SG, Clayton A, Bao W, Guico-Pabia CJ: Post hoc analysis of the efficacy and safety of desvenlafaxine 50 mg/day in a randomized, placebo-controlled study of perimenopausal and postmenopausal women with major depressive disorder. Menopause 2014;21:799-806.

30 Macias-Cortes ED, Llanes-Gonzalez L, Aguilar-Faisal L, Asbun-Bojalil J: Is metabolic dysregulation associated with antidepressant response in depressed women in climacteric treated with individualized homeopathic medicines or fluoxetine? The HOMDEP-MENOP Study. Homeopathy 2017;106:3-10.

3. Inclusion of participants not around time of menopause (n=1)

31 Burt VK, Wohlreich MM, Mallinckrodt CH, Detke MJ, Watkin JG, Stewart DE: Duloxetine for the treatment of major depressive disorder in women ages 40 to 55 years. Psychosomatics 2005;46:345-354.

4. Lack of adequate outcome measurement or baseline data for analysis (n=3)

32 van Lith ND, Motke JC: Opipramol in the climacteric syndrome. A double-blind, placebo-controlled trial. Maturitas 1983;5:17-23.

33 Kornstein SG, Pedersen RD, Holland PJ, Nemeroff CB, Rothschild AJ, Thase ME, Trivedi MH, Ninan PT, Keller MB: Influence of sex and menopausal status on response, remission, and recurrence in patients with recurrent major depressive disorder treated with venlafaxine extended release or fluoxetine: analysis of data from the PREVENT study. The Journal of clinical psychiatry 2014;75:62-68.

34 Estrella RE, Landa AI, Lafuente JV, Gargiulo PA: Effects of antidepressants and soybean association in depressive menopausal women. Acta Pol Pharm 2014;71:323-327.

5. Combination therapy, not just antidepressant (n=1)

35 Caan B, LaCroix AZ, Joffe H, Guthrie KA, Larson JC, Carpenter JS, Cohen LS, Freeman EW, Manson JE, Newton K, Reed S, Rexrode K, Shifren J, Sternfeld B, Ensrud K: Effects of estrogen and venlafaxine on menopause-related quality of life in healthy postmenopausal women with hot flashes: a placebo-controlled randomized trial. Menopause 2015;22:607-615.

**Supplementary Table S3.** Summary of main adverse event in the included studies in the current meta-analysis

| Author, year^Ref^ | Treatment | D/O (%) | Top reason for D/O | S/E (%) | Most common adverse events | Serious AE |
| --- | --- | --- | --- | --- | --- | --- |
| Davari-Tanha, 2016^29^ | Venlafaxine 75 mg/d  Citalopram 20 mg/d  Placebo | 0.0%  0.0%  0.0% | Nil  Nil  Nil | Nil  Nil  Nil | Vomiting (30%), Nausea (25%)^*^, constipation (40%)  Nausea (40%)^*^, lethargy (40%), vomiting (35%)  Headache (15%), lethargy (15%), Nausea (5%)^*^ | Nil  Nil |
| Macias-Cortes, 2015^12^ | Fluoxetine 20 mg/d  Placebo | 15.2%  14.0% | Nil  Nil | Nil  ^**^Nil | Anxiety (17.4%), headache (13%), dizziness (10.9%)  Fatigue (14%), headache (14%) | Nil |
| Clayton, 2013^11^ | Desvenlafaxine 50 mg/d  Placebo | 14.7%  18.0% | AE  Ineffective | 71.4%  68.2% | Headache (15.2%), nausea (11.1%)  Headache (11.5%) | ^***^2  ^***^2 |
| Cheng, 2013^16^ | Desvenlafaxine 100 mg/d  Desvenlafaxine 150 mg/d  Placebo | 12.4%  16.4%  ^**^9.8% | AE  AE  AE | Nil  Nil  Nil | Nil  Nil  Nil | Nil  Nil  Nil |
| Kornstein, 2010^30^ | Desvenlafaxine 100-200 mg/d  Placebo | 17.2%  12.8% | AE  Patient request | 85.2%  75.2% | Dry mouth (24%), nausea (18.6), somnolence (15%)  Nausea (12%), dry mouth (10%), somnolence (7%) | 3  2 |
| Soares, 2008^31^ | Paroxetine 25 mg/d  Placebo | 3.6%  17.9% | AE  Ineffective | Nil  Nil | Nil  Nil | Nil  Nil |
| Suvanto-Luukkonen, 2005^17^ | Fluoxetine 20 mg/d  Citalopram 30 mg/d  Placebo | 34.0%  32.0%  ^**^40.0% | Ineffective  Ineffective  Ineffective | Nil  Nil  Nil | Dry mouth (54%), nausea (9%)  Dry mouth (39%), nausea (9%)  Dry mouth (36%), nausea (13%) | Nil  Nil  Nil |

Abbreviation: AE: adverse events; D: day; D/O: drop-out; EPT: estrogen plus progestogen therapy; S/E: side effect

^*^Statistically significant

^**^Not statistically significant

^***^Unrelated to treatment

**Supplementary Table S4.**  Assessment of methodological quality of studies included in the current meta-analysis using Cochrane risk of bias tool

| Study (year) ^Ref^ | Random sequence | Allocation concealment | Performance bias | Detection bias | Attrition bias | Reporting bias | Other bias |
| --- | --- | --- | --- | --- | --- | --- | --- |
| Davari-Tanha  (2016) ^29^ | Low | Unclear | Low | Low | Low | Low | Low (N) |
| Macias-Cortes (2015) ^12^ | Low | Low | Low | Low | Low | Low | Low (N) |
| Clayton  (2013) ^11^ | Low | Unclear | Low | Low | Unclear | Low | Low (N) |
| Cheng  (2013) ^16^ | Unclear | Unclear | Low | Low | Unclear | Low | High (X) |
| Kornstein  (2010) ^30^ | Low | Unclear | Low | Low | Low | Low | High (X) |
| Soares  (2008) ^31^ | Unclear | Unclear | Low | High | Unclear | Low | High (X) |
| Suvanto-Luukkonen (2005) ^17^ | Low | Unclear | Low | Low | High | Low | High (X) |

N, received no funding from pharmaceutical companies; X, received funding from pharmaceutical companies
